# Circulating bacterial DNA in cardiovascular disease

**DOI:** 10.3389/fcvm.2025.1476165

**Published:** 2025-04-15

**Authors:** Sarah Appleby, Rachel Purcell

**Affiliations:** ^1^ Department of Medicine, Christchurch Heart Institute, University of Otago Christchurch, Christchurch, New Zealand; ^2^ Department of Surgery, University of Otago Christchurch, Christchurch, New Zealand

**Keywords:** circulating bacterial DNA, cbDNA, cell-free DNA, cardiovascular disease, biomarker, liquid biopsy

## Abstract

Cardiovascular disease (CVD) remains a global health burden despite advances in prevention and treatment. Conventional biomarkers, while effective for a number of patient groups, fail to provide personalized diagnosis and prognosis, necessitating the exploration of novel markers. Advancements in sequencing technology have unveiled the role of cell-free DNA (cfDNA) as a reservoir of genetic information from all cells within the body, and associations between elevated cfDNA levels and CVD risk factors and status have been reported. Recent attention has turned to a subset of cfDNA, circulating bacterial DNA (cbDNA), derived from gut microbiota, as a potential biomarker. Investigations into microbial translocation from the gut, particularly the phenomenon of ‘leaky gut,’ reveal its association with CVD and provide a potential source for cbDNA. Here, we review the existing literature on cbDNA in CVD, highlighting its potential diagnostic and prognostic value. Current studies have largely been carried out in small, disparate cohorts, using different sample types and a range of methodologies. While cbDNA shows potential as a diagnostic and prognostic biomarker, the lack of consensus in methodologies and populations studied calls for standardized approaches and large cohorts to establish cbDNA as a reliable CVD biomarker. Future research should focus on identifying the source of cbDNA and its pathological relevance, utilizing advanced sequencing techniques and standardized cohorts for conclusive findings.

## 1 Introduction

**Figure 1:**
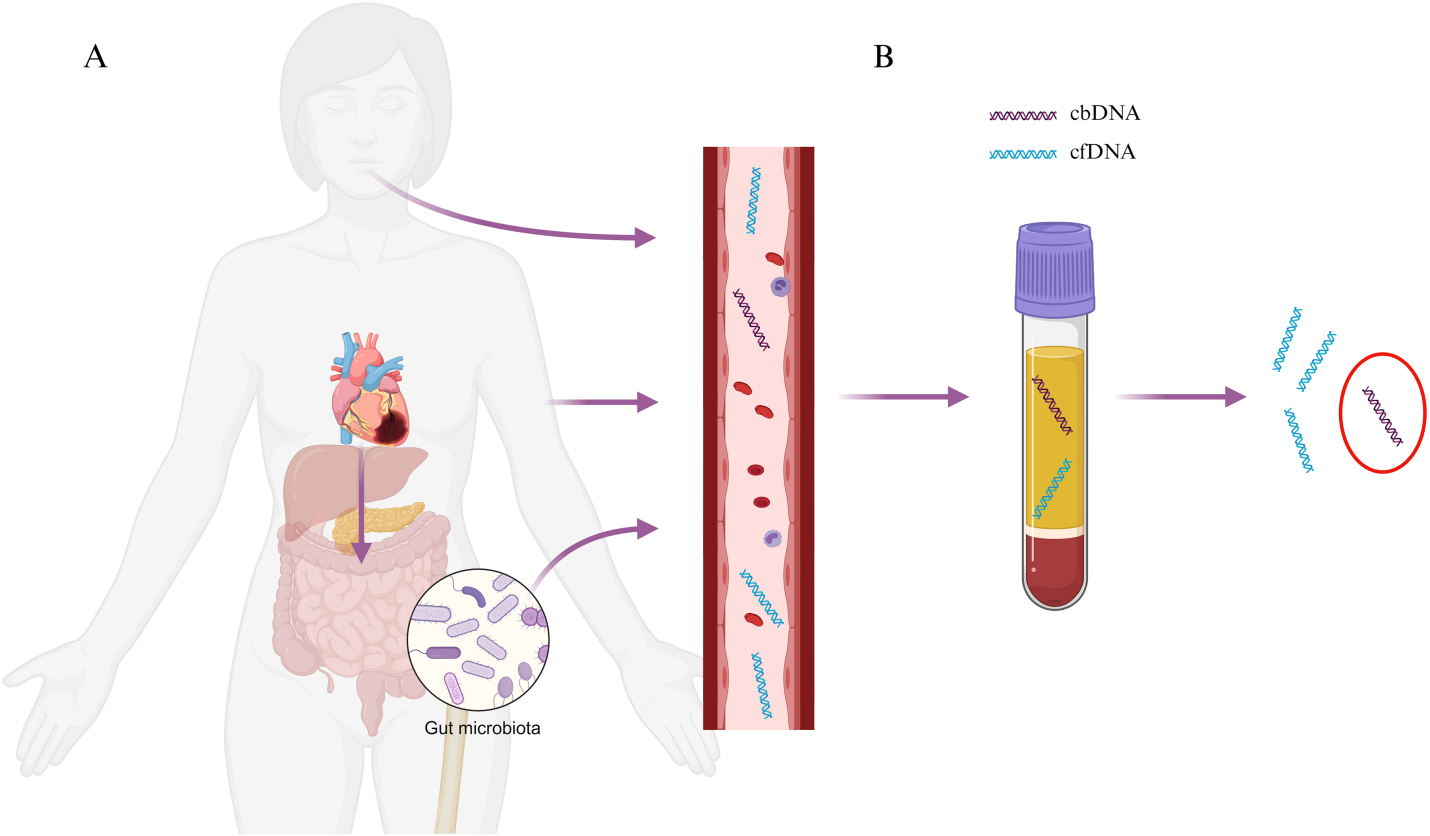
Graphical depiction of **(A)** potential routes of translocation of bacterial DNA into the bloodstream including oral, skin, gut and **(B)** subsequent detection in plasma samples. Created with BioRender.com.

Despite medical advances, cardiovascular disease (CVD), remains a leading cause of morbidity and mortality worldwide, consuming a significant amount of health-care resources (1). The complexity of the disease means that although a number of patients benefit from the current biomarkers, some are being disadvantaged by this ‘one size fits all’ testing. Accordingly, the identification of novel biomarkers is required to complement or replace current markers to allow for timely diagnosis and improve clinical outcomes for patients and reduce the large numbers of people affected by the disease. Recent advances in sequencing technology have led to the explosion of knowledge in the field of circulating cell-free DNA (cfDNA). cfDNA is found in bodily fluids and acts like a genetic reservoir that carries genetic information from all cells within the body. The amount of cfDNA is minimal in healthy individuals but concentrations have been shown to increase with age, pregnancy, exercise and disease (2), thus, making it an attractive biomarker target for clinical applications. Several recent studies have shown a strong association between the presence of cfDNA with both the risk factors and status of CVD (3, 4). Although research into cfDNA is increasing, there are very few studies identifying and characterizing circulating bacterial DNA (cbDNA), a subset of cfDNA derived from gut bacteria. As a result, cbDNA in CVD is not well understood, nor is its biomarker utility. Here, we review the current literature on cbDNA in CVD (Figure 1 and Table 1).

**Table 1:** Published studies of circulating bacterial DNA in CVD populations or with CVD outcomes.

Samples	Methodology	Sample type	Main findings	Study
3,936 general population	Amplicon sequencing of bacterial 16S rRNA	Peripheral blood leukocytes	Participants with the lowest Eubacteria levels and highest Proteobacteria had a higher risk of incident CVD events.	Amar et al. (25)
13 VHD, 11 IHD, 7 CHD (31 CVD), 10 controls	Amplicon sequencing of V3 region for bacterial 16S rRNA	Whole blood	CVD patients had increased Proteobacteria compared to healthy controls.	Rajendhran et al. (26)
100 STEMI, 50 stable CHD*, 49 controls	Amplicon sequencing of the V4 region of bacterial 16S rRNA	Peripheral blood leukocytes	Greater microbial diversity and increased gut specific bacteria (*Lactobacillus, Bacteroides, Streptococcus)* in STEMI patients.	Zhou et al. (18)
99 MI, 103 controls with high CVD risk	Amplicon sequencing of V3-V4 region of bacterial 16S rRNA	Whole blood	Increased circulating bacterial abundance in MI patients compared to non-MI. Specific bacterial signature in MI patients but lower diversity than controls.	Amar et al. (27)
24 VHD, 30 IHD, 26 CHD (80 CVD), 40 controls	16S rRNA/β-globin ratio by PCR Metagenomic shotgun sequencing (3 CVD, 3 controls)	Plasma	Higher relative levels of cbDNA β-globin ratio in CVD patients compared to controls. CVD patients had increased Proteobacteria compared to healthy controls.	Dinakaran et al. (28)
17 end-stage renal disease, with matched controls	Amplicon sequencing of V3-V4 region of bacterial 16S rRNA	Serum	Greater Actinobacteria and less Proteobacteria observed in cases than in controls. Proportion of Actinobacteria and Proteobacteria was slightly associated with risk of cardiovascular death.	Sumida et al. (29)
191 Peritoneal dialysis patients	PCR amplification of bacterial 16S rRNA	Plasma	Each doubling in cbDNA concentration confers a 26.9% excess in risk of developing a composite cardiovascular end point.	Szeto et al. (30)

CVD, cardiovascular disease; VHD, valvular heart disease; IHD, ischemic heart disease; CHD, congenital heart disease; STEMI, ST-segment elevation myocardial infarction; CHD*, coronary heart disease; MI, myocardial infarction; cbDNA, circulating bacterial DNA.

## 2 Gut-heart axis

In addition to established risk factors, there is an expanding body of evidence to support the role of the human microbiome and the gut-heart axis in the establishment of cardiovascular disease (CVD) (5). The gut microbiome consists of trillions of microbes, including bacteria, viruses and eukaryotes, which has the ability to modify or influence numerous biological functions in the host, including contributing to the pathogenesis of multiple immune-mediated diseases and metabolic conditions (6). There is increasing evidence to show that gut dysbiosis, the adverse changes in the gut microbiome present, can induce inflammation, leading to the onset of chronic disease (7). In the context of CVD, products of microbial metabolism, for example trimethylamine-N-oxide (TMAO), have been shown to promote atherogenic activity (8). Research into the gut-heart axis has led to growing interest in the potential of gut health interventions, such as dietary modifications, prebiotics and probiotics as a means of improving cardiovascular health (5).

## 3 Leaky gut

Historically, blood was presumed to be sterile and the identification of microbes in the circulatory system was only thought to occur in infectious disease and sepsis (9, 10), but of late both microbes and cbDNA has been found in both healthy individuals and patients with various diseases (11–14). Unlike the proposed mechanisms of active release of cfDNA into the circulation from human cells, namely, apoptosis or necrosis, an alternate hypothesis has been proposed for the origin of cbDNA. There are several suggested routes for microbial ingress into the circulation including oral (15) and skin, however, microbial translocation from the gut has been proposed as the major contributor of cbDNA in the circulation (16). Here, failure of the gut barrier, termed “leaky gut”, enables bacteria and their endotoxins to cross the intestinal barrier into the circulatory system. Increased intestinal permeability has been observed in animal models of myocardial infarction (MI) (17, 18) and atherosclerosis (19), as well as human studies of CVD including chronic heart failure (20), MI (18), atherosclerosis and coronary artery disease (21, 22). Markers of gut bacterial translocation into the circulatory system, namely lipopolysaccharide (LPS) and D-lactate, have also been linked to CVD prognosis. Zhou et al. provided the first evidence that CVD outcomes post-MI are associated with intestinal microbiota translocation into the systemic circulation (18). They reported that the plasma markers LPS and D-lactate, were significantly increased and correlated with systemic inflammation and predicted the occurrence of a first major adverse cardiovascular event.

## 4 Methodological differences

Initial studies identifying circulating microbes in CVD focused on single bacterial species detected using PCR (23, 24). With the advent of microbiome analysis by 16S rRNA gene amplicon sequencing and/or metagenomic analysis, the detection of all microbes/microbial DNA in the circulation could be identified, allowing for microbial diversity and taxa abundance to be assessed. A number of studies have now been published that utilize 16S rRNA gene amplicon sequencing; the 16S rRNA gene contains highly conserved regions interspersed with hypervariable regions that provide genus/species-specific signatures that are used for bacterial taxonomic profiling. Utilizing metagenomic sequencing or direct DNA sequencing increases the taxonomic resolution. However, unlike culture-based techniques, these methods do not differentiate between live and dead bacteria, making it difficult to infer cause or consequence. Further, the choice of whole blood, plasma or serum when analyzing circulating bacterial DNA generates different results.

## 5 cbDNA in whole blood

Utilizing 16S rRNA sequencing of whole blood, Amar et al. were the first to show that microbial dysbiosis in the circulation may play a role in the pathological processes in CVD (25), in a large (*n* = 3,936) longitudinal study of the general population, free of obesity and diabetes. They found that baseline Eubacteria was significantly lower and Proteobacteria higher in peripheral blood leucocytes of patients that went on to have an acute cardiovascular event comprising MI, myocardial ischemia and/or significant coronary stenosis. Using multivariate Cox model analysis, they found a 3.7-fold increase in the risk of CVD events in participants who had the lowest levels of Eubacteria and the highest levels of Proteobacteria (*p* = 0.003) (25). Similarly, an increase in Proteobacteria has also been observed in the whole blood of CVD patients (mix of valvular, ischemic and congenital heart disease) compared to healthy controls in a small study in an Indian population (26).

Elevated bacterial abundance and specific bacterial signatures have been observed in the whole blood of patients following an MI. In ST-segment elevation myocardial infarction (STEMI, *n* = 100) patients, one study found greater microbial diversity when compared to patients with stable coronary heart disease (CHD, *n* = 50) and healthy individuals (*n* = 49) (18). Blood bacterial characteristics were distinct for each group, with an increased abundance of gut specific bacteria (*Lactobacillus, Bacteroides*, and *Streptococcus*) found in STEMI patients (18). This finding was not replicated by Amar et al., who reported that although acute MI patients (*n* = 99) had higher abundance and a specific bacterial signature, they had lower blood bacterial diversity compared to non-MI individuals at high CVD risk but free of coronary disease (*n* = 103) (27).

## 6 cbDNA in plasma

Few studies have analyzed cbDNA in plasma, with only one comparison being made between whole blood (26) and plasma (28). Here circulating bacterial DNA was analyzed in the same group of CVD patients (valvular, ischemic and congenital heart disease). In plasma, patients with CVD were found to have higher circulating bacterial diversity compared to healthy individuals. Actinobacteria and Proteobacteria were the two major phyla represented in both CVD and control samples, followed by a smaller fraction of Firmicutes. Actinobacteria was dominant in CVD patients, followed by Proteobacteria, whereas the opposite was observed in the controls (28). Although these results are based on a small subset of the cohort with metagenomic shotgun sequencing with low depth. These results were in contrast to what was reported by the same group in whole blood (26) where patients had an increased amount of Proteobacteria and a reduction in Firmicutes in comparison to healthy controls. Analysis of the circulating bacteria in whole blood was performed using 16S rRNA amplicon sequencing, whereas only relative abundance was assessed in the plasma of patients by PCR. These methodological differences may account for some of the differences seen. Further, Dinakaran et al. were the first to report the use of bacterial abundance in plasma to investigate the diagnostic utility of cbDNA in CVD. The authors showed that increased bacterial abundance was able to discriminate ischemic heart disease (*p* < 0.0001) and CHD patients (*p* < 0.0001) from healthy controls, but the sample size was small and highly selected.

## 7 cbDNA as a prognostic marker

The prognostic utility of cbDNA has been investigated in patients with kidney disease [end stage renal disease (29) and peritoneal dialysis patients (30)], a group who have an elevated risk of developing CVD or having a CVD event. A small pilot study of 17 hemodialysis patients who subsequently died from a CVD event and matched controls, reported that the proportion of cbDNA from Actinobacteria and Proteobacteria present in serum was slightly associated with risk of cardiovascular death [adjusted odds ratios [95% confidence intervals] = 1.12 [0.98–1.29] and 0.88 [0.76–1.02] for 1% increase, respectively] (29). Another study of 191 new peritoneal dialysis patients (30) showed a weak negative correlation between cbDNA abundance and the number of hospital admissions and duration of hospitalization for cardiovascular reasons. They reported that each doubling in plasma cbDNA concentration confers a 26.9% excess in risk of developing a composite cardiovascular end point comprising a number of outcomes for example, cardiovascular death, non-fatal MI or stroke and hospital admission for unstable angina. When cbDNA level was split into quartiles, the event-free survival for those with the lowest cbDNA concentration was 86.1% whereas those with the highest was 30.8% (30).

## 8 Ethnic variation in cbDNA

While some studies suggest that cfDNA abundance and characteristics might vary across different ethnic groups (31, 32), there has been no research to date exploring these differences in cbDNA. Diet, lifestyle, genetic lineage, socioeconomic status, and metabolic profiles are all factors that may differ between ethnic groups and could potentially contribute to varying cbDNA profiles. Among the studies reviewed here, only one provided demographic information regarding the ethnic composition of the studied population. Sumida et al. reported that their population was predominantly African American (70.6%), with 23.5% White and 5.9% other. No formal comparisons were made, but they only had a small number of total participants (*n* = 34). Understanding ethnic differences in cbDNA profiles would provide valuable insights into how genetic and environmental interactions within the gut-heart axis influence CVD, and future studies should aim to include a wide range of ethnic populations in order to ensure any findings are widely applicable.

## 9 Discussion

Currently, there are very few studies investigating cbDNA in CVD. Each study utilizes different methodologies and sample types across ethnically diverse populations with varying CVD profiles, making comparisons difficult. As such, there is no consensus as to abundance, diversity, origin and specific bacterial signatures when comparing CVD patients to healthy individuals. The use of PCR to assess cbDNA, particularly without adequate controls, is not a gold standard method of analyzing the microbiome and studies using this method are not reliable. Further, the accuracy of 16S rRNA amplicon sequencing in plasma remains to be determined. cbDNA in plasma is highly fragmented and 16S rRNA primer binding sites may not be available. This will vastly underestimate bacterial DNA abundance and potentially the diversity in a sample. Future studies should employ DNA sequencing techniques to assess cbDNA in the plasma by either whole metagenomic or direct sequencing. There is an accumulating amount of research showing that a major source of cbDNA in the circulation is arising from leaky gut. Future research should identify the source of the cbDNA and the pathological relevance. While there is potential in investigating cbDNA as a biomarker in CVD, more research using large standardized cohorts and methodologies needs to be performed.
